# A novel transformer-based DL model enhanced by position-sensitive attention and gated hierarchical LSTM for aero-engine RUL prediction

**DOI:** 10.1038/s41598-024-59095-3

**Published:** 2024-05-02

**Authors:** Xinping Chen

**Affiliations:** College of Artificial Intelligence and Big Data, Chongqing College of Electronic Engineering, Chongqing, 401331 China

**Keywords:** RUL prediction, Transformer encoder, Aero-engine, Attention, LSTM, Electrical and electronic engineering, Mechanical engineering

## Abstract

Accurate prediction of remaining useful life (RUL) for aircraft engines is essential for proactive maintenance and safety assurance. However, existing methods such as physics-based models, classical recurrent neural networks, and convolutional neural networks face limitations in capturing long-term dependencies and modeling complex degradation patterns. In this study, we propose a novel deep-learning model based on the Transformer architecture to address these limitations. Specifically, to address the issue of insensitivity to local context in the attention mechanism employed by the Transformer encoder, we introduce a position-sensitive self-attention (PSA) unit to enhance the model's ability to incorporate local context by attending to the positional relationships of the input data at each time step. Additionally, a gated hierarchical long short-term memory network (GHLSTM) is designed to perform regression prediction at different time scales on the latent features, thereby improving the accuracy of RUL estimation for mechanical equipment. Experiments on the C-MAPSS dataset demonstrate that the proposed model outperforms existing methods in RUL prediction, showcasing its effectiveness in modeling complex degradation patterns and long-term dependencies.

## Introduction

Accurate prediction of remaining useful life (RUL) is crucial for proactive maintenance, reducing casualties and economic losses. RUL prediction methods are classified into physics-based, artificial intelligence-based, and hybrid models^1–7^. Physics-based methods use specific models based on failure mechanisms to explain degradation patterns and integrate real-time monitoring data for RUL assessment. However, they face limitations in complex mechanical systems^8–11^. Artificial intelligence methods learn degradation patterns from observational data without expert knowledge. They excel in predicting complex systems where physical or statistical models are inadequate and have gained attention with advancements in technology^12^. Hybrid methods integrate the advantages of different approaches but may be limited in complex rotating machinery^13^.

With the accumulation of valuable data and the rapid advancement of computing power, deep learning (DL) has become a hot topic and has been successfully applied in various engineering fields. DL + PHM has gained popularity in both academia and industry. For instance, in the early days, some methods employed classical RNN models for regression tasks on time series data. However, RNN models face challenges such as the gradient vanishing or exploding problem^14^, limiting their performance in long sequence prediction tasks. As a solution, RNN variants like LSTM^15,16^ and GRU^17^ emerged, which use nonlinear gating mechanisms to control the flow of information and alleviate these limitations to some extent. The research on using gated networks for RUL prediction has been growing rapidly. Zhang et al.^18^ proposed an LSTM-Fusion network structure for estimating the RUL of aircraft engines. This network integrates observation sequences of different lengths to extract hidden information effectively. Miao et al.^19^ introduced a novel dual-task stacked LSTM method that simultaneously evaluates the degradation stages and predicts the RUL of aircraft engines. Liu et al.^20^ presented a multi-level prediction approach for aircraft engine health using LSTM and statistical process analysis for bearing fault prediction. Zhang et al.^21^ proposed a dual-task network structure based on bidirectional GRU and a mixture of multiple gating expert units. This structure enables simultaneous evaluation of aircraft engine health status and prediction of the RUL. Ma et al.^22^ introduced a new prediction model based on deep wavelet sequence gated recurrent units for RUL prediction of rotating machinery. The proposed wavelet sequence gated recurrent units generate wavelet sequences of different scales through a wavelet layer. Xiao et al.^23^ enhanced the robustness of the BLSTM model for RUL prediction by adding Gaussian white noise to the health indicators based on principal component analysis. Song et al.^24^ constructed aircraft engine health indicators using variational autoencoders and employed the BLSTM model for RUL prediction.

In addition to enhancing the model's temporal data processing capability using recurrent approaches, another alternative is the use of convolutional neural networks (CNNs), which employ shared receptive fields to improve spatial feature extraction^25^. CNN-based models have also been successfully applied in RUL prediction and have shown competitive performance. Zhu et al.^26^ proposed a multi-scale CNN for predicting the RUL of bearings. Compared to traditional CNNs, this network maintains synchronization of global and local information. Li et al.^27^ introduced a new approach based on deep CNNs for RUL prediction using raw data. Yang et al.^28^ employed a dual CNN model for RUL prediction. In this model, the first CNN model identifies early fault points, while the second CNN model predicts the RUL. Jiang et al.^29^ transformed time series data into multi-channel data and used CNN to construct health indicators, leading to improved accuracy in residual life prediction.

The Transformer model^30–32^, as one of the most popular deep learning architectures in recent years, has been introduced for sequence data modeling. It efficiently handles long sequences of parallel data and can be applied to time series data of varying lengths. It has achieved remarkable success in various industrial applications, including natural language processing^33^, machine vision^34^, medical diagnosis^35^, and more. In recent years, it has also been gradually applied in the field of RUL prediction. Zhang et al.^36^ introduced a novel Transformer-based bidirectional self-attention deep model for RUL prediction. This method is a fully self-attention-based encoder-decoder structure without any RNN/CNN modules. Su et al.^37^ proposed an adaptive Transformer that combines attention mechanisms and recurrent structures for predicting the RUL of rolling bearings. It directly models the relationship between shallow features and RUL, mitigating the vanishing gradient problem and better representing complex time degradation patterns. Based on the proposed shared temporal attention layer, Chadha et al.^38^ developed two Transformer models specifically designed for handling multivariate time series data and applied them to predict the RUL of aircraft engines. Chang et al.^39^ proposed a novel Transformer model for RUL prediction based on a sparse multi-head self-attention mechanism and knowledge distillation technique. It effectively reduces the computational burden of the model and improves domain adaptation capability for raw signal data of rolling bearings. Ren et al.^40^ introduced a T2 tensor-assisted multiscale Transformer model to accurately predict the RUL of industrial components. Ding et al.^41^ presented a new convolutional Transformer model capable of extracting degradation-related information from both local and global original signals.

In this study, we propose a DL model based on a Transformer-based auto-encoder for the task of RUL prediction. Unlike RNN and CNN models, the Transformer architecture allows for the processing of a sequence of data in a single pass by leveraging attention mechanisms, enabling access to any part of the historical data without being limited by distance. This makes it potentially more powerful in capturing long-term dependencies. However, the adopted dot-product self-attention in Transformers results in the extracted high-level features being insensitive to their local context at each time step^34^, which requires the model to invest more effort in estimating the corresponding RUL. Therefore, we introduce position-aware self-attention units (PSA) to enhance the model's ability to focus on the positional relationships of the input data at each time step and improve the incorporation of local context. Additionally, to leverage the improved features extracted by the encoder, we design a gated hierarchical long short-term memory network (GHLSTM) for regression predictions at different time scales, further enhancing the accuracy of RUL prediction for mechanical equipment. The main contributions in the article are as follows.

1. The traditional attention mechanism used in the Transformer encoder is insensitive to the local context, which is essential for predicting remaining useful life. The proposed position-aware self-attention (PSA) mechanism captures the positional relationships of input data, enabling the model to incorporate local context and generate more effective hidden features. This leads to improved accuracy in predicting remaining useful life.
2. For enhancing the ability to model long-term dependencies and improve performance in handling large-scale sequential data, the gated hierarchical long short-term memory (GHLSTM) network is proposed, which learns features at different time scales, enables regression predictions at multiple scales, and provides comprehensive feature learning. This results in improved accuracy in predicting RUL.
3. Experimental results on a widely used aerospace dataset demonstrate the superiority of our proposed method over other existing methods based on quantitative evaluation metrics.

The outline of the article is as follows. Section "Introduction" provides an introduction to the research topic. Section "Theoretical basis" presents the theoretical basis. Section "Proposed methodology" gives a detailed deduction of the proposed DL model. Section "Experimental analysis" is the content of experiments and relevant analysis. And finally, a conclusion is given in Section "Conclusion".

## Theoretical basis

The Transformer was first introduced in 2017 for NLP tasks^42^. It is a sequence-to-sequence model that essentially functions as an auto-encoder, composed of a sophisticated encoder module and a decoder module. The encoder module maps the input sequence to a high-dimensional hidden vector, which is then fed into the decoder to generate the output sequence. Unlike recurrent networks with their sequential data input nature, the Transformer is capable of capturing long-term dependencies by utilizing self-attention mechanisms based on dot products. Transformer-based models have achieved remarkable performance in various time series tasks, including natural language processing, computer vision, and PHM.

**Figure 1:**
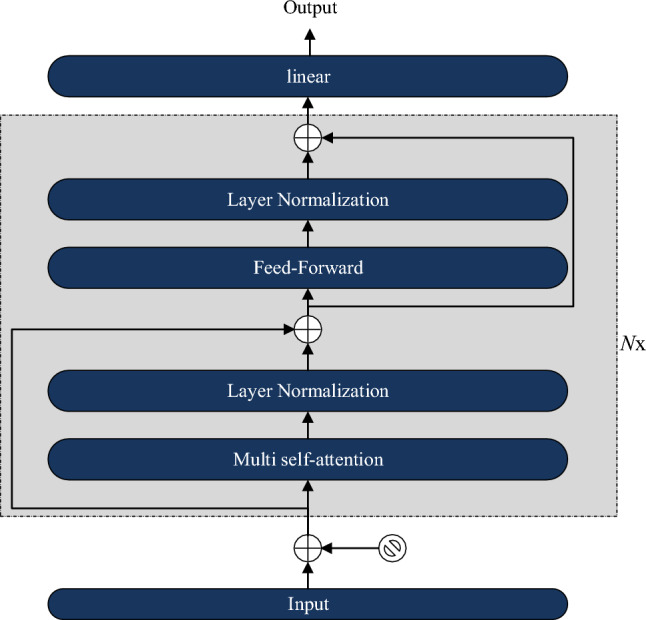
The diagram of the Transformer encoder module.

The proposed model primarily focuses on the improved structure of the Transformer encoder module. Therefore, in this section, we provide a detailed explanation of the main components and architecture of the Transformer encoder module. The Transformer encoder structure, as shown in Fig. 1, mainly consists of multi-head attention, feed-forward networks, and position encoding.

### Multi-head self-attention

The multi-head self-attention mechanism is a variant of the attention mechanism widely used in natural language processing and machine translation tasks. It is an extension of the self-attention mechanism designed to enhance the modeling capacity of the model for different semantic information. The self-attention mechanism allows the model to interact and exchange information between different positions in the input sequence, while the multi-head self-attention mechanism further expands this interactive capability. It achieves this by applying the attention mechanism to different projections in multiple subspaces, creating multiple attention heads. Each attention head has its own set of parameters and can learn different attention weights to capture the associations between different semantic information.

The calculation process of the multi-head self-attention mechanism is as follows. We project the input sequence, i.e. ${\textbf{f}} = \left\{ {f_{i} } \right\}_{i = 1}^{t}$ with $f_{i}$ *w.r.t* $x_{i}$ and $f_{i} \in R^{d}$, into multiple subspaces through linear transformations. For each attention head, we use different parameter matrices to perform the projection, obtaining representations for each sub-space. We denote the parallel attention calculations as *H*, which represents the multi-head attention mechanism:

1 $$MultiHead({\textbf{Q, K, V}}) = C{\text{on}}cat(\{ head_{j} \}_{j = 1}^{H} ){\textbf{W}}^{A} ,$$

2 $$head_{j} = Attention({\textbf{Q, K, V}})_{j} = soft\max (\frac{{{\textbf{Q}}_{j} {\textbf{K}}_{j}^{T} }}{{\sqrt {d_{k} } }}){\textbf{V}}_{j} ,$$

3 $$\begin{gathered} {\textbf{K}}_{j} = {\textbf{fW}}_{j}^{k} \hfill \\ {\textbf{V}}_{j} = {\textbf{fW}}_{j}^{v} \hfill \\ {\textbf{Q}}_{j} = {\textbf{fW}}_{j}^{q} \hfill \\ \end{gathered}$$

where ${\textbf{W}}^{A} \in {\textbf{R}}^{{H_{{d_{k} \times d}} }}$ and $d_{k} = d/H$;${\textbf{k}}_{j}$,${\textbf{V}}_{j}$ and ${\textbf{Q}}_{j}$ are the key, value and query vectors; ${head}_{j}$ is the *j*th attention head; ${\textbf{W}}_{j}^{k}$, ${\textbf{W}}_{j}^{v}$, ${\textbf{W}}_{j}^{q} \in {\textbf{R}}^{{d \times d_{k} }}$ are the trainable matrixes.

### Feedforward neural network and position encoding

The feed-forward NN is composed of two full connection (FC) layers with ReLU activation function, whose formula is as follows,

4 $$F({\textbf{x}}) = {\textbf{W}}_{2} \cdot {\text{Re}} LU({\textbf{W}}_{1} {\textbf{x}} + {\textbf{b}}_{1} ) + {\textbf{b}}_{2} ,$$

where ${\textbf{W}}$ and ${\textbf{b}}$ are the weights and bias of the following connected FC layers,${\textbf{x}}$ is the input of the forward neural network.

The formula of position encoding is demonstrated as follows,

5 $$\begin{gathered} p_{i}^{(2s)} = \sin (i/10000^{2s/d} ) \hfill \\ p_{i}^{(2s + 1)} = \cos (i/10000^{2s/d} ) \hfill \\ \end{gathered}$$

By the above design, for given input with any length l, $p_{i}$ and $p_{i + l}$ has a linear relationship, which helps the regression model learn the sequence relationship effectively. Thus the final input of the transformer encoder module is ${\textbf{X}} = {\textbf{x}} + {\textbf{p}}$.

## Proposed methodology

### The proposed enhanced Transformer model

**Figure 2:**
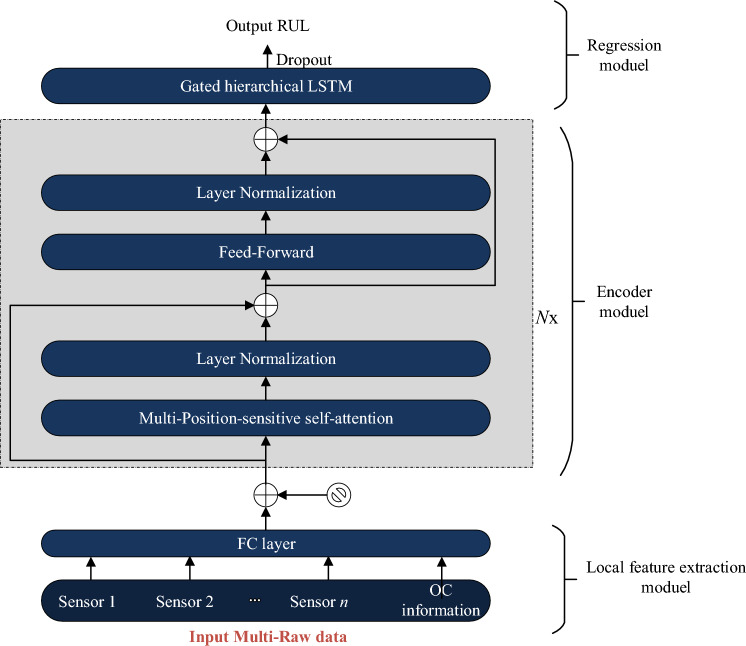
The proposed enhanced Transformer model.

The proposed enhanced Transformer model consists of three parts: the feature extraction module, the encoding module, and the regression module, as shown in Fig. 2. The feature extraction module consists of a simple fully connected (FC) layer and position encoding, which performs a simple non-linear dimensionality reduction on the multidimensional raw data and incorporates positional information. The encoding module further compresses and extracts valuable latent features from the extracted features. Compared to the encoding module of traditional Transformers, the proposed model mainly adopts Position-Sensitive Attention (PSA) to replace the self-attention component, enabling the encoding module to capture more contextual information. The PSA unit is integrated to address the insensitivity to local context in the Transformer encoder, thus enhancing the model's ability to incorporate positional relationships and local context at each time step. PSA collectively contributes to the generation of latent features with higher efficacy, which benefits the remaining useful life prediction in the regression module. The regression module utilizes the proposed GHLSTM with multiple hidden features at different time scales for regression prediction. Compared to ordinary linear regression or recursive network-based regression, it can more effectively learn from hidden features, thereby improving the accuracy of RUL predictions.

Supposed that for each sample *i*, the predicted RUL is $\overline{Rul}_{i}$ and the true RUL is $Rul_{i}$. Mean square error (MSE) is adopted as a loss function to tune the learnable parameters $\theta$ of the proposed enhanced Transformer model during the training stage by the optimization Adam, whose formula is given below,

6 $$L\left( {MSE,\theta } \right) = \frac{1}{2}\sum\limits_{i = 1}^{N} {\left( {\overline{Rul}_{i} - Rul_{i} } \right)^{2} } .$$

Table 1 shows the hyper-parameters of the proposed DL method. The optimized hyper-parameters of the model are obtained by the grid search. The pseudocode of the proposed prediction method has been summarized in Table 2.

**Table 1:** The hyper-parameters of the proposed enhanced Transformer model.

Sub layer	Hyperparameter value	Sub layer	Hyperparameter value
Linear	14	Number of encoder module	2
MPSA	4	Learning rate	0.005
Feedforward	128	Output layer	1
GHLSTM	15	Dropout	0.2

**Table 2:**
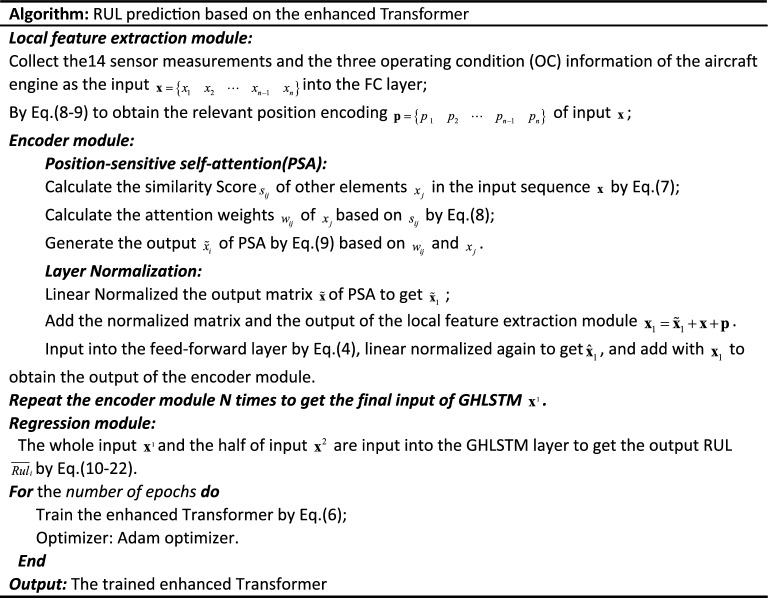
The pseudocode of the proposed RUL prediction method.

### Position-sensitive self-attention (PSA)

To overcome the issue of insensitivity of high-level features to local context in Transformer encoders, we introduce a position-aware self-attention (PSA) unit in our proposed model. This improvement enables the model to focus on the positional relationships of input data at each time step, thereby enhancing its capability to capture local context. Consequently, this approach computes similarity scores between each input element and all other elements, considering both content and positional encodings. Attention weights are then computed based on these scores, and the output is formed by taking the weighted sum of the inputs. By incorporating positional information, the PSA mechanism enhances the model's ability to capture local context, leading to more accurate attention weights and improved feature representations, generating more effective hidden features for accurately predicting the RUL of mechanical equipment. This enhanced sensitivity to local context is crucial for accurately predicting RUL. The deduction of PSA is described as follows.

(1) Construction of the input:

The input of PSA consists of the input sequence $${\textbf{x}} = \left\{ {\begin{array}{*{20}c} {x_{1} } & {x_{2} } & {\begin{array}{*{20}c} \cdots & {x_{n - 1} } \\ \end{array} } & {x_{n} } \\ \end{array} } \right\}$$ and the relevant position encoding $${\textbf{p}} = \left\{ {p\begin{array}{*{20}c} {_{1} } & {p_{2} } & {\begin{array}{*{20}c} \cdots & {p_{n - 1} } \\ \end{array} } & {p_{n} } \\ \end{array} } \right\}$$, where $x_{i}$ is the feature representation of the* i*th element, and $p_{i}$ is the position encoding of the position *i* whose formulas are Eq. (5).

(2) The calculation of the similarity score:

For each element $x_{i}$ in the input sequence, calculate the similarity Score $s_{ij}$ of other elements $x_{j}$ in the input sequence ${\textbf{x}}$, meantime considering the influence of position encoding $p_{ij}$, thus the formula is deduced as follows,

7 $$\begin{aligned} s_{{ij}}^{1} &= similarity\left( {x_{{_{i} }} \;\;\; p_{{_{{ij}} }} } \right) = \left( {x_{{_{i} }} \cdot p_{{_{{ij}} }} } \right)/\left( {\left\| {x_{{_{i} }} } \right\|*\left\| {p_{{_{{ij}} }} } \right\|} \right) \\ s_{{ij}}^{2} &= similarity\left( {x_{{_{i} }} \;\;\; p_{{_{{ij}} }} } \right) = \left( {x_{{_{i} }} \cdot p_{{_{{ij}} }} } \right)/\left( {\left\| {x_{{_{i} }} } \right\|*\left\| {p_{{_{{ij}} }} } \right\|} \right) \\ s_{{ij}} &= similarity\left( {s_{{ij}}^{1} \;\;\;s_{{ij}}^{2} } \right) = \left( {s_{{ij}}^{1} \cdot s_{{ij}}^{2} } \right)/\left( {\left\| {s_{{ij}}^{1} } \right\|*\left\| {s_{{ij}}^{2} } \right\|} \right) \end{aligned}$$

(3) The calculation of attention weights:

For each element $x_{i}$, calculating the attention weights $w_{ij}$ based on the similarity Score,

8 $$w_{ij} = soft\max \left( {{\text{s}}_{ij} } \right) = \frac{{\exp \left( {{\text{s}}_{ij} } \right)}}{{\sum\limits_{k = 1}^{n} {\exp \left( {{\text{s}}_{ik} } \right)} }}.$$

(4) Finally, the output element $\tilde{x}_{i}$ after PSA is the weighted sum of attention weights with $w_{ij}$ the input element,

9 $$\tilde{x}_{i} = \sum\limits_{j = 1}^{n} {w_{ij} .x_{j} } .$$

Position-sensitive attention mechanism considers both the correlation between elements and the influence of position encoding, resulting in more accurate and position-aware attention weights.

### Gated hierarchical long short-term memory network (GHLSTM)

**Figure 3:**
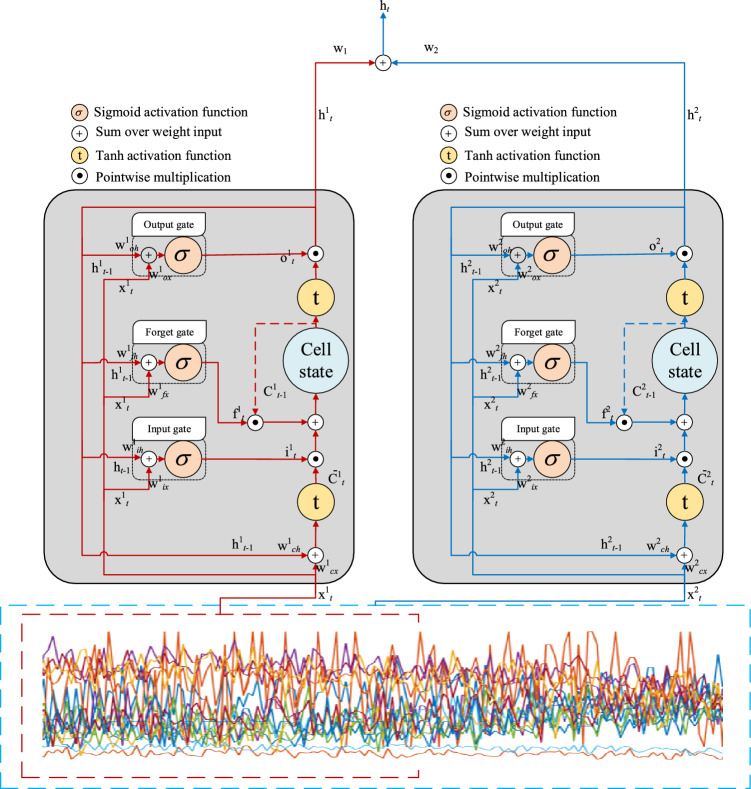
The structure of the proposed GHLSTM.

The goal of the Hierarchical LSTM with gating is to further enhance the LSTM model's ability to model long-term dependencies and improve its performance in handling large-scale sequential data. It achieves this by introducing multiple levels of gating to gradually model dependencies at different time scales. The diagram of GHLSTM is shown in Fig. 3. The GHLSTM network is designed to model long-term dependencies across multiple time scales, thereby enhancing the accuracy of RUL prediction. This method consists of two hierarchical LSTM layers: a top-level LSTM for modeling global long-term dependencies and a bottom-level LSTM for capturing medium-term dependencies. The top LSTM processes the entire input sequence to capture long-term dependencies. The bottom LSTM processes half of the input sequence to capture medium-term dependencies. The outputs of the top and bottom LSTMs are concatenated to form the final output as a comprehensive temporal representation. The approach enables the model to adaptively focus on relevant features across different time scales, thereby improving the overall RUL prediction accuracy.

For the top hierarchical LSTM, the whole sequence of the input ${\textbf{x}}_{1t}$ is input into the LSTM cellular, the formula is,

10 $${\textbf{i}}_{1t} = \sigma \left( {{\textbf{w}}_{1ix} {\textbf{x}}_{1t} + {\textbf{w}}_{1ih} {\textbf{h}}_{1t - 1} + {\textbf{b}}_{1i} } \right),$$

11 $${\textbf{f}}_{1t} = \sigma \left( {{\textbf{w}}_{1fx} {\textbf{x}}_{1t} + {\textbf{w}}_{1fh} {\textbf{h}}_{1t - 1} + {\textbf{b}}_{1f} } \right),$$

12 $${\textbf{o}}_{1t} = \sigma \left( {{\textbf{w}}_{1ox} {\textbf{x}}_{1t} + {\textbf{w}}_{1oh} {\textbf{h}}_{1t - 1} + {\textbf{b}}_{1o} } \right),$$

13 $${\overline{\textbf{c}}}_{1t} = \tanh \left( {{\textbf{w}}_{1cx} {\textbf{x}}_{1t} + {\textbf{w}}_{1ch} {\textbf{h}}_{1t - 1} + {\textbf{b}}_{1c} } \right),$$

14 $${\textbf{c}}_{1t} = {\textbf{f}}_{1t} \odot {\textbf{c}}_{1t - 1} + {\textbf{i}}_{1t} \odot {\overline{\textbf{c}}}_{1t} ,$$

15 $${\textbf{h}}_{1t} = {\textbf{o}}_{1t} \odot \tanh \left( {{\textbf{c}}_{1t} } \right),$$

For the bottom hierarchical LSTM, half of the whole sequence of the input ${\textbf{x}}_{2t}$ is input into the LSTM cellular to extract the hidden feature in another time scale. Noted that the time scale can be deiced by the requirements. The formula of the bottom hierarchical LSTM cellular is,

16 $${\textbf{i}}^{2}_{t} = \sigma \left( {{\textbf{w}}^{2}_{ix} {\textbf{x}}^{2}_{t} + {\textbf{w}}^{2}_{ih} {\textbf{h}}^{2}_{t - 1} + {\textbf{b}}^{2}_{i} } \right),$$

17 $${\textbf{f}}_{2t} = \sigma \left( {{\textbf{w}}_{2fx} {\textbf{x}}_{2t} + {\textbf{w}}_{2fh} {\textbf{h}}_{2t - 1} + {\textbf{b}}_{2f} } \right),$$

18 $${\textbf{o}}_{2t} = \sigma \left( {{\textbf{w}}_{2ox} {\textbf{x}}_{2t} + {\textbf{w}}_{2oh} {\textbf{h}}_{2t - 1} + {\textbf{b}}_{2o} } \right),$$

19 $${\overline{\textbf{c}}}_{2t} = \tanh \left( {{\textbf{w}}_{2cx} {\textbf{x}}_{2t} + {\textbf{w}}_{2ch} {\textbf{h}}_{2t - 1} + {\textbf{b}}_{2c} } \right),$$

20 $${\textbf{c}}_{2t} = {\textbf{f}}_{2t} \odot {\textbf{c}}_{2t - 1} + {\textbf{i}}_{2t} \odot {\overline{\textbf{c}}}_{2t} ,$$

21 $${\textbf{h}}_{2t} = {\textbf{o}}_{2t} \odot \tanh \left( {{\textbf{c}}_{2t} } \right),$$

where ${\textbf{w}}_{ix}$(${\textbf{w}}_{fx}$, ${\textbf{w}}_{ox}$ and ${\textbf{w}}_{cx}$), and ${\textbf{w}}_{ih}$(${\textbf{w}}_{fh}$, ${\textbf{w}}_{oh}$, and ${\textbf{w}}_{ch}$) are the input and recurrent matrix weights, ${\textbf{b}}_{i}$(${\textbf{b}}_{f}$, ${\textbf{b}}_{o}$, and ${\textbf{b}}_{c}$) are the bias of the hidden layer,${\overline{\textbf{c}}}_{t}$ denotes the internal state of the cell, ${\textbf{c}}_{t}$ denotes the memory cell state, $\sigma$ represents the sigmoid function; $\tanh$ represents the tanh activation, and $\odot$ represents the pointwise multiplication. And the definitions of the two LSTM cellular are the same.

Then the output of the top hierarchical LSTM cellular ${\textbf{h}}_{1t}$ and the bottom hierarchical LSTM cellular ${\textbf{h}}_{2t}$ are combined to construct the final output of GHLSTM ${\textbf{h}}_{t}$,

22 $${\textbf{h}}_{t} = {\textbf{w}}_{1} {\textbf{h}}_{1t} + {\textbf{w}}_{2} {\textbf{h}}_{2t} .$$

where ${\textbf{w}}$ are the connected weights making the two outputs the same dimension.

## Experimental analysis

### Evaluation indexes

The widely used evaluation indexes for RUL prediction, i.e. score and root mean square error (RMSE), are adopted for the quantitated demonstration of the model performance. And the formulas of the indexes are given below,

23 $$A_{i} = \left\{ {\begin{array}{*{20}c} {\exp ( - ((\overline{{Rul_{i} }} - Rul_{i} )/13)) - 1,} \\ {\exp ((\overline{{Rul_{i} }} - Rul_{i} )/10) - 1,} \\ \end{array} } \right.\begin{array}{*{20}c} {\begin{array}{*{20}c} {} & {} \\ \end{array} \overline{{Rul_{i} }} < Rul_{i} } \\ {\begin{array}{*{20}c} {} & {} \\ \end{array} \overline{{Rul_{i} }} \ge Rul_{i} } \\ \end{array}$$

24 $$Score = \sum\limits_{i = 1}^{N} {A_{i} } ,$$

25 $$RMSE = \sqrt {\frac{1}{N}\sum\limits_{i = 1}^{N} {\left( {Rul_{{\text{i}}} - \overline{{Rul_{i} }} } \right)^{2} } } ,$$

**Figure 4:**
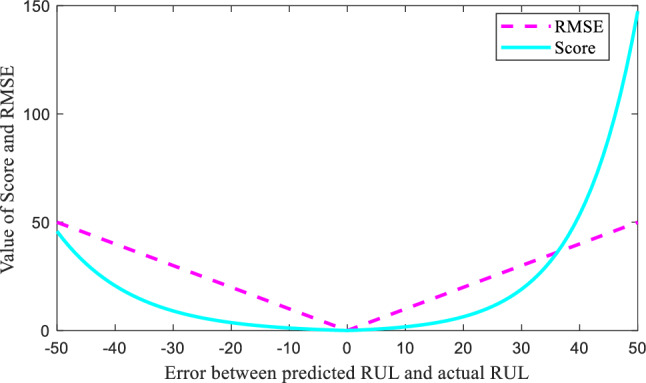
The curves of the two evaluation indexes.

As shown in Fig. 4, it serves as a graphical representation of the trend of evaluation metrics. The curve's changing trend is easily discernible from the graph. When the error is positive, the Score value increases rapidly, indicating that the Score imposes a stronger penalty on lagged predictions. This characteristic aligns better with practical engineering requirements. Therefore, the Score is considered more reasonable compared to RMSE.

### The description of the C-MAPSS dataset

**Figure 5:**
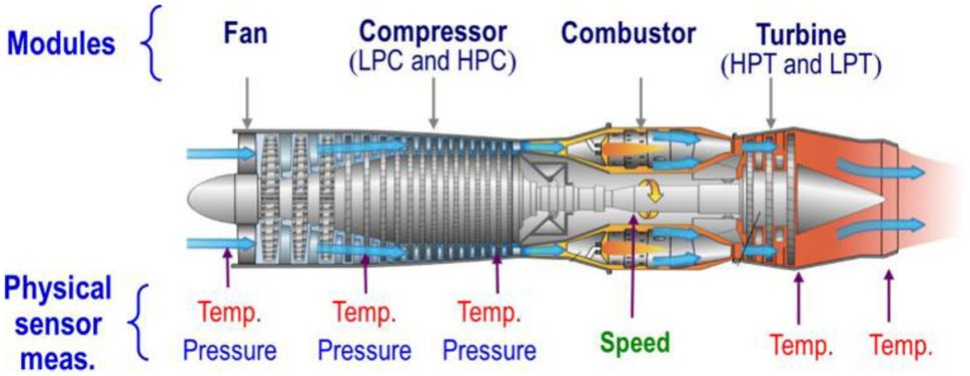
Diagram of the aircraft engine^27^.

To demonstrate the effectiveness and superiority of the proposed method in predicting the remaining useful life of aircraft engines, we utilized the C-MAPSS dataset provided by NASA^27^, whose diagram is shown in Fig. 5. The dataset consists of a collection of aircraft engines, as shown in the figure. Furthermore, to showcase the capabilities of the proposed method under different operating conditions and fault modes, we used the simplest FD001 dataset and the most complex FD004 dataset as validation data.

FD001 dataset consists of 100 engines operating under a single operating condition and a single fault mode. The engines have varying lifespans, with the shortest operational cycle being 128 and the longest being 362. The dataset includes sensor measurements, such as fan speed, compressor speed, oil pressure, and various temperatures, along with operational settings like throttle setting and true airspeed. FD004 is a more complex dataset derived from the same aircraft engine simulations, containing 249 engines operating under 6 different operating conditions and experiencing 2 different fault modes. Similar to FD001, the engines have lifespans ranging from 128 to 543 operational cycles. The sensor measurements and operational settings are also similar to FD001, but the inclusion of multiple conditions and fault modes makes FD004 significantly more challenging for RUL prediction. The data details are presented in Table 3. The tasks of FD001 and FD004 remain the same, to accurately predict the RUL of each engine.

**Table 3:** The details of dataset C-MAPSS.

Subset	FD001	FD004
Total number of engines	100	249
Operating condition	1	6
Type of fault	1	2
Maximum cycles	362	543
Minimum cycles	128	128

### The preprocessing of input

**Figure 6:**
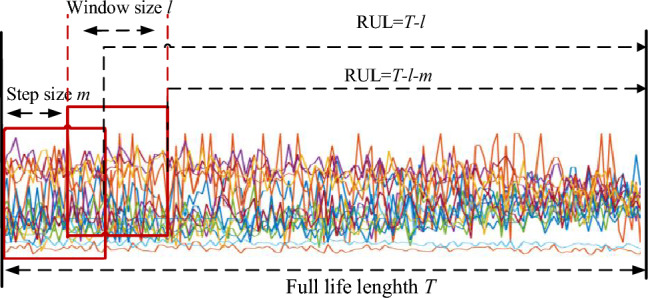
Processing of data segmentation.

Firstly, we delete the unimportant sensor measurements (sensors 1, 5, 6, 10, 16, 18, and 19), which are stable and have less degradation information. According to the literature^27^, operating condition information is also helpful in RUL prediction. Thus the final input matrix consists of the remaining 14 sensor measurements and the three operating condition information. The second step, data segmentation is executed, as shown in Fig. 6 For the ith input with n dimension input and l sequence length (window size), the relevant RUL label is set as Ts–l–(i–1) × m, where m and T are the sliding steps and full-lifecycle value. Through greedy search by the experiments, the hyper-parameters l and m are set to 30 and 1. The last step is the linear piecewise RUL preprocessing for the RUL label $Rul_{\max } = 125$ as below,

26 $$Rul = \left\{ {\begin{array}{*{20}c} {Rul,{\text{ if }}Rul \le Rul_{\max } \, } \\ {Rul_{\max } ,{\text{ if }}Rul > Rul_{\max } \, } \\ \end{array} } \right.$$

### The analysis and comparison of RUL prediction results

#### RUL prediction performance of the proposed method

**Figure 7:**
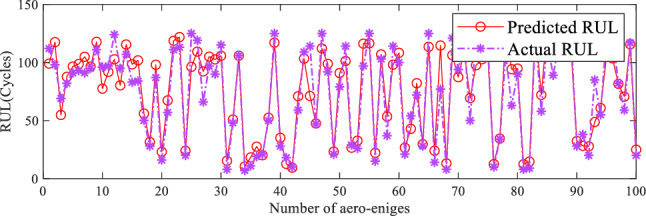
RUL prediction performance on FD001.

**Figure 8:**
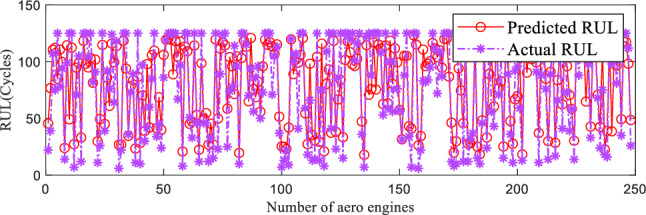
RUL prediction performance on FD004.

The predicted results of the proposed model on the FD001 and FD004 subsets are shown in the figures below. In Figs. 7, 8, the value of the x-axis denotes the test engine number of the subset, while the y-axis represents the remaining useful life values (in cycles). The predicted remaining useful life and the actual remaining useful life of the test engines are represented by the red solid line and the purple dashed line, respectively. Overall, the predicted remaining useful life values of the test set engines in both subsets roughly align with the actual values, indicating the effectiveness of the proposed method in predicting the remaining useful life in these two subsets. Additionally, the error between the predicted life and the actual life in Fig. 7 is smaller than in Fig. 8. This indicates that the proposed model performs better on the FD001 subset compared to the FD004 dataset. This is because the degradation trend of the aerospace engine under a single operating condition is relatively simpler. Moreover, there is a significant overlap between the degradation trends of the training set and the test set of aerospace engines. Therefore, the proposed method achieves higher accuracy in predicting the remaining useful life of aerospace engines under a single operating condition and a single fault compared to complex operating conditions and compound faults.

**Figure 9:**
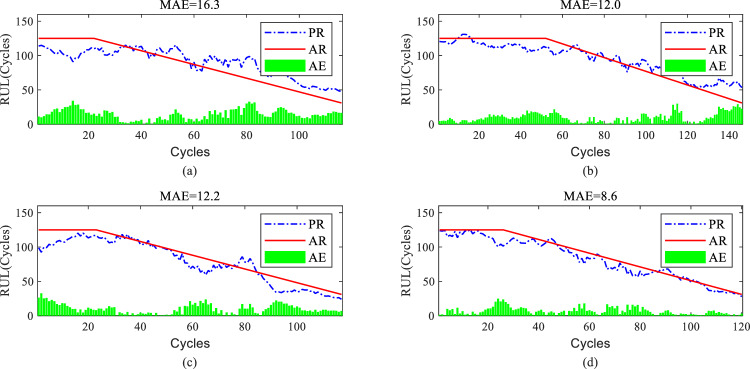
RUL prediction performance of engines of FD001 ((**a**) # 46, (**b**) # 58, (**c**) # 66, and (**d**) # 92).

**Figure 10:**
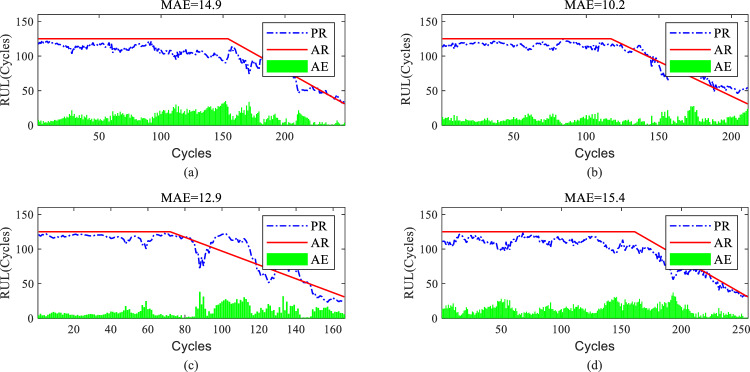
RUL prediction performance of engines of FD004 ((**a**) # 35, (**b**) # 68, (**c**) # 100, and (**d**) # 151).

The proposed model's predictions on the complete degradation process are shown in Figs. 9a–d and 10a–d for four randomly selected aerospace engine engines from each subset The predicted RUL (PR) and actual RUL (AR) are represented by the blue line and red line, the absolute error (AE), calculated by based on PR and AR at each time instant, is denoted by the green bar. Thus the average error is represented by the mean of all AE values (MAE). The overall remaining useful life prediction results for FD001 are significantly better than for FD004, as indicated by the average MAE value. As the number of cycles increases, the degradation trend of aerospace engine engines becomes apparent. The proposed model exhibits higher accuracy in predicting the remaining useful life of most aerospace engine engines in the later stages of degradation compared to the earlier stages, as shown in Figs. 9a, c, d and 10a–d.

#### Ablation experiments

To validate the superiority of the proposed method, namely the effectiveness of PSA and GHLSTM, a series of erosion experiments were conducted. Assuming model m1 represents the proposed enhanced Transformer model, model m2 uses the same model architecture except for the attention part, which adopts the traditional multi-head self-attention module. Similarly, model m3 employs the same deep learning module except for the regression module, which uses the traditional LSTM model. Model m4 also uses the same deep learning module, but both its regression module and self-attention module adopt traditional models. All models are fine-tuned and ten parallel experiments are conducted. The mean values and standard deviation (std) value of RMSE and score across all experiments are calculated, as shown in Table 4. The mean value is taken as the final predicted RUL, while std is used to quantify the robustness of the RUL prediction. It is evident from the table that the proposed model exhibits the lowest metric values and demonstrates the best predictive performance compared to other models. The std value is significantly lower than the mean value. Moreover, the predictive performance of m2 and m3 is superior to that of m4. These observations indicate that the proposed techniques contribute to the improvement of the accuracy in predicting the RUL.

**Table 4:** The RUL prediction comparisons of different methods on subset FD001and FD002.

Model	FD001		FD004	
	Score	RMSE	Score	RMSE
m4 (mean ± std)	301 ± 33	14.58 ± 0.41	2310 ± 199	16.35 ± 0.63
m3 (mean ± std)	265 ± 28	13.45 ± 0.35	1765 ± 165	15.89 ± 0.54
m2 (mean ± std)	244 ± 23	13.65 ± 0.28	1580 ± 136	15.76 ± 0.42
m1 (mean ± std)	220 ± 23	13.14 ± 0.21	1420 ± 125	14.25 ± 0.25

#### Compared with state-of-arts

To further highlight the advantages of the proposed enhanced Transformer in predicting remaining useful life, a comparative experiment was conducted between the proposed model and several state-of-the-art methods^42–49^. To provide a comprehensive evaluation, the training set and testing set are fixed as the same for all compared models, each model was fine-tuned with the optimization goal of maximizing the accuracy in predicting remaining useful life, and 10 parallel experiments were conducted on FD001 and FD004 subsets. Subsequently, the scores and RMSE values based on the prediction results of all the aforementioned methods are listed in Table 5. From the table, it can be observed that all methods perform best in the FD001 subset and worst in the FD004 subset. This is because FD001 has the simple operating condition and fault type, while FD004 is the most complex subset with a larger number of tested engines.

**Table 5:** The RUL prediction comparisons of different methods on subset FD001and FD002.

Model	FD001		FD004	
	Score	RMSE	Score	RMSE
MONBNE^42^	334	15.04	6558	28.66
LSTM + attention + handscraft feature^43^	322	14.53	5649	27.08
Acyclic graph network^44^	229	11.96	3370	22.43
AEQRNN^45^	N/A	N/A	4597	20.60
MCLSTM-based^46^	260	13.21	2926	22.10
SMDN^47^	240	13.72	1591	18.24
SIGRNNDWI^48^	229	13.14	1568	15.12
MSBLS^49^	–	–	1785	17.75
Proposed (mean ± Std)	220 ± 23	13.14 ± 0.21	1420 ± 125	14.25 ± 0.25
Improvement	4%	–	10%	6%

On the FD001 dataset, which contains a single operating condition and fault mode, the proposed model achieved a Score of 220 ± 23 and an RMSE of 13.14 ± 0.21. This performance showed an improvement of 4% in Score compared to the best-performing existing method (acyclic graph network), which obtained a Score of 229 and an RMSE of 11.96. While the RMSE value of the proposed method is lower than acyclic graph network, the evaluation index Score is more in line with the actual engine and the Score value is lower than Acyclic Graph Network. This means that the comprehensive performance of the proposed method is best compared with other models.

Furthermore, on the more complex FD004 dataset, which encompasses multiple operating conditions and faults, the proposed model achieved a Score of 1420 ± 125 and an RMSE of 14.25 ± 0.25. This performance demonstrated an improvement of 10% in Score and 6% in RMSE compared to the best-performing existing method (SIGRNNDWI), which obtained a Score of 1568 and an RMSE of 15.12. Overall, the proposed model exhibited improved RUL prediction accuracy on both datasets, particularly on the more complex FD004 subset. These results validate the effectiveness of the proposed PSA and GHLSTM techniques in enhancing RUL prediction for aircraft engines.

## Conclusion

For accurately predicting the RUL of aero-engines, this article proposed a novel enhanced transformer-based DL method with the PSA mechanism and GHLSTM method. The main contributions of the article are as follows. One is the proposed PSA mechanism, PSA can solve the problem of the traditional attention mechanism that the extracted high-level features are insensitive to their local context at each time step. Another is the development of GHLSTM, GHLSTM can learn the hidden features at different time scales, which helps to improve RUL. The effect of the proposed technologies has been validated by the ablation experiments. Through the quantitative evaluation of common indicators, the proposed method has an average improvement of 7% in Score and 11% in RMSE compared with other methods on the RUL prediction tasks of FD001 and FD004.

## Data Availability

The datasets used and/or analyzed during the current study are available from the corresponding author upon reasonable request.
